# Cardiovascular Adaptations to Four Months Training in Middle-Aged Amateur Long-Distance Skiers

**DOI:** 10.3390/diagnostics10070442

**Published:** 2020-06-30

**Authors:** Natalia Grzebisz

**Affiliations:** Vistula School of Hospitality, Faculty of Dietetics, 02-787 Warsaw, Poland; n.grzebisz@gmail.com; Tel.: +48-22-457-23-00

**Keywords:** cardiovascular capacity, performance, cross-country skiing amateur, heart

## Abstract

Cross-country skiing has a positive effect on health. However, without an individual, thoughtful, and professional plan, it can cause irreversible health problems from overload and injury. The impact of exercise on results is well understood within the group of professional athletes. However, this remains unknown within the group of amateur cross-country skiers and marathon runners—in particular, the impact of the summer preparation period in which training loads performed in the oxygen zone combined with resistance training dominate. The aim of this study was to assess changes in the cardiovascular capacity and body mass composition of male cross-country skiers in the preparation period of their macrocycle. Variables were analyzed using basic descriptive statistics: mean and standard deviation (SD). To compare the results from both measurements (initial and final) the paired Wilcoxon test was used. A statistically significant increase was noted in maximum oxygen uptake and maximum minute ventilation, and a decrease in body fat content, maximum lactate concentration and lactate threshold, and heart rate on anaerobic threshold. Research indicated that in the amateur group increases similar to those in top competitors were achieved in the parameters tested, but the initial level was often significantly lower.

## 1. Introduction

Monitoring of physical effort in competitive sport is a key element in the training process used by trainers, physiologists, and doctors. It also allows to assess the health and capacity of amateurs. In particular, the determination of cardiovascular capacity parameters establishes the initial level before training, allows to assess the progress made and to predict the negative impact of excessive physical exertion. This applies in particular to endurance efforts such as long-distance cross-country skiing. It requires many years of physical activity and the use of periodization of training, for example, to avoid overloading and its negative impact on health.

The parameter used to assess exercise capacity in endurance athletes is the maximum oxygen uptake (VO_2max_). Its level and change during many years of training is very well described in the literature when it comes to professional athletes. However, there are no reports on the impact of physical effort on the level and change of VO_2max_ in amateur long-distance skiing, which significantly differs in terms of requirements from other sports.

In recent years long-distance skiing (especially the Ski Classics series) has gained the attention of scientists, but the research has been focused on elite skiers [1]. To my knowledge, this paper is the very first on amateur long-distance skiers and their cardiovascular capacity.

The majority of popular long-distance races (30 up to 220 km) are easy in terms of track profile (flat sections and slight climbs, easy downhills), but this type of effort is long-term and characterized by near-threshold intensity. It requires not only efficiency, but also good technique [2].

Cross-country skiing is one of the most demanding sports. In addition to the high energy demand, the specificity of the effort is also important. It involves all body muscles, especially the upper parts [3,4].

This discipline has a positive effect on health. Scientists emphasize the role of systematic physical activity in improving the cardiovascular risk profile and body weight composition in middle-aged men. In the population low body mass index and high body fat percentage are associated with increased mortality. Therefore, qualitative and not quantitative changes in the body are important. In addition, body fat content should be within the reference ranges. This may bring greater cardiovascular benefits than a decrease in body mass index with a decrease in muscle mass [5]. 

However, without an individual, thoughtful, and professional plan, it can cause irreversible health problems from overload and injury. This is why it is so important to be aware of the body and to control the impact of training. An additional advantage of monitoring is a better adaptation of the training plan and the prevention of fatigue and overtraining. The monitoring process should apply to both athletes and amateurs. Both groups exert submaximal and maximum effort during competition, but professional athletes have a coaching team that checks for negative changes in the body [6].

Amateurs have to take care of this themselves, in terms of physiotherapy, physiological monitoring, biochemistry, diet, supplementation, training loads, as well as monitoring progress. Their work and private lives also have a significant impact on these changes. The impact of exercise on results is well understood within the group of professional athletes. However, it remains unknown within the group of amateur cross-country skiers and marathon runners—in particular, the impact of the summer preparation period, in which training loads performed in the oxygen zone combined with resistance training dominate. The use of roller skis is also unique, as it not only involves almost all muscles in the body but also relieves the joints [7].

The aim of this study was to assess changes in the cardiovascular capacity of cross-country skiers in the preparation period of their macrocycle (the annual cycle of preparations of athletes).

## 2. Materials and Methods 

### 2.1. Subjects

The study was conducted in accordance with the guidelines of Good Clinical Practice and the Helsinki Declaration. The study was approved by the Bioethics Committee at the Faculty of Human Nutrition and Consumption at Warsaw University of Life Sciences (SGGW) (Nr. 38p/2018, approved on 22 January 2019). All subjects and parents gave their written consent before any testing. The research group consisted of 16 well-trained amateur skiers (mean age 37.5 +/− SD years, and 37.9 in the second test). The competitors worked professionally in a big city and could spend up to 90 min daily for physical training. There were no data on cardiovascular risk factors and changes in response to training for this group.

The research was carried out at the end of the transitional period in May and after the preparation period in September. The criteria for inclusion in the study were: consent to participate in the study, possession of current medical approval, and completion of at least three long-distance races in the last season. Exclusion criteria were: lack of consent for participation in the study, poor health (any disease occurrence), or lack of medical consent.

### 2.2. Anthropometric Measurements

Body weight was measured on the Tanita MC-980 MA Plus Body Composition Analyzer, consisting of an eight-point touch electrode system. The test was carried out just before the incremental exercise tests began. The following were determined: body weight, water content, minerals, vitamins, fat content in the body (% and kg) and slim mass muscle (muscle mass in % and kg), WHR (waist-to-hip ratio), and BMI (body mass index). All measurements were carried out at Sportslab Sports Diagnostics Center in Warsaw, Poland.

### 2.3. Measurement of Aerobic Capacity (VO_2max_ Test)

To assess the aerobic capacity expressed by the level of maximum oxygen uptake (VO_2max_) a time trial test was used with gradually increasing intensity. This test was performed on a treadmill using HP Cosmos CPET equipment and Cosmed Quark/k4B2. The test started at a speed of 6 km/h and a 0% treadmill inclination. Then every 3 min, the speed was increased by 1 km/h, and the inclination by 1%. The test was continued until the subjective feeling of exhaustion by the competitor (to refuse). The frequency of heart contractions at rest and during exercise was recorded using the Garmin ANT+ heart rate monitor. The paper presents the maximum results of the test below. Dr. Müller Super GL Analyser was used to measure lactate concentrations. All measurements were carried out at Sportslab Sports Diagnostics Center in Warsaw, Poland.

### 2.4. Training Loads

For four months, the skiers performed a systematic endurance and strength effort. It consisted of running, resistance training in the gym, cycling, and, above all, targeted training, which was on roller skis. Individual exercise zones were designated for each person during the first exercise studies.

**Figure 1:**
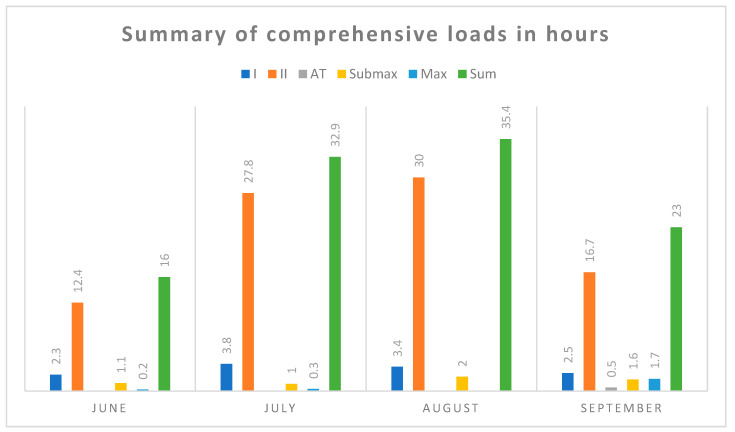
Monthly summary of comprehensive loads in hours.

**Figure 2:**
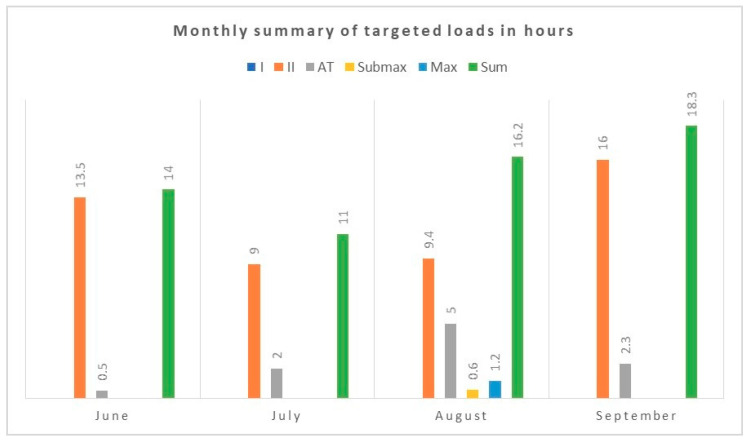
Monthly summary of targeted loads in hours.

**Figure 3:**
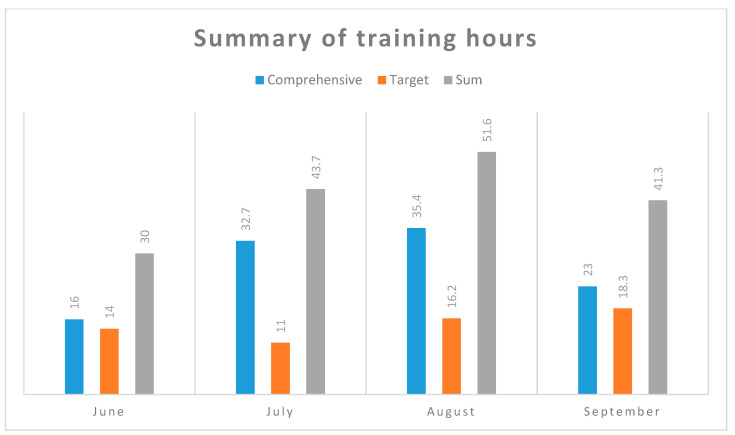
Summary of training hours.

Figure 1, Figure 2 and Figure 3 summarize the comprehensive efforts (running, cycling, swimming, general development exercises) and targeted training (ski imitation, roller skis, Ercolina, which is the machine for strengthening the arms and upper body, and special exercises). These data contain information about the intensity and volume of hourly training during the test period. The intensity was pre-rated in five exercise zones: I, II-aerobic, AT (anaerobic threshold)-mixed, anaerobic-Submaximal (Submax), and maximal (Max).

### 2.5. Statistics

Variables were analyzed using basic descriptive statistics: arithmetic mean and standard deviation (SD). To compare the results from both measurements (initial and final) the paired Wilcoxon test was used, taking a materiality level of 0.05. The value of 0.05 was assumed as the significance level (denoted by * *p* < 0.05, * *p* < 0.01, *** *p* < 0.001).

## 3. Results

A comparison of the results of the body weight and exercise parameters of the first and second tests is presented in Table 1. Statistically significant changes were recorded in the: percentages of body fat mass, body fat mass (kg), VO_2max_ (relative) (mL/kg/min), maximum lactate concentration (mmol/L), maximum ventilation (L/min), lactate concentration (on the threshold) AT (mmol/L), and breathing rate on AT (bpm). The other parameters did not show statistically significant changes (see Table 1).

## 4. Discussion

The main findings of this study are: (1) a statistically significant change in relative VO_2max_ (*p* = 0.008), similar to that in elite cross-country skiing; (2) a change in body fat mass (in absolute terms as well as in %); (3) a change in maximum ventilation.

In the study, the largest statistically significant change was recorded in relative VO_2max_ (*p* = 0.008). This corresponded to an increase of 2.1 mL/kg/min, which was 4.3% on a percentage basis. There are a lack of publications on the impact of a training program (especially roller ski training) on performance parameters in amateur long-distance skiing that could be used to compare these changes. However, we can compare the results to research on other endurance disciplines, e.g., triathlon. A group of 32 participants followed a training program lasting five months with the goal to finish the Half-Ironman competition (which is a long-distance event, similar in hours of effort to 90 km of skiing) [8]. The program significantly increased the maximal oxygen consumption of participants (45.9 ± 8.2 to 48.6 ± 7.5 mL/kg/min, *p* = 0.002) and the difference between tests was 5.9%. In elite cross-country skiing the change in relative VO_2max_ was slightly lower (3.1 ± 4.5%) in response to loads in the preparation mesocycle [9]. 

These results confirm that regular physical activity increases the values of VO_2max_, which is the main indicator of the fitness level. Many other studies have also confirmed the positive effects of regular training on VO_2max_ for people over 30 years of age. These effects were greater in the cases of people who led sedentary lifestyles, as well as in cases of introducing HIIT (high intensity interval training) to the training program [10]. Furthermore, high-intensity exercise may reduce by up to 50% the decline in VO_2max_ in young and middle-aged men if the activity is maintained long term. It is worth noting that age-related loss of VO_2max_ is 10% [11]. Researchers indicated that in the elite the changes occurred between the early, middle, and late preparation phases of the macrocycle with minor changes in the competitive season [12]. It can be assumed that this period of preparation in amateurs also significantly affects health-related changes in the body.

It is worth emphasizing that studies did not show significant differences between the results of the time-to-exhaustion test in running and roller skiing. Elite cross-country skiers did not elicit higher VO_2max_ during roller ski skating than during running, and this relationship did not change during the pre-season training period. This may be a monitoring tip for trainers and physiologists, for whom the use of roller skis on a treadmill is limited or impossible.

Several studies have demonstrated that male and female world-class skiers are among the endurance athletes with the highest VO_2max_. Accordingly, world-class performance has been associated with maximal values above 70 and 80 mL/kg/min, or 4.0 and 6.0 L/min, in female and male skiers, respectively [13]. Values of VO_2max_ [14] in a group of athletes (55.46 mL/kg/min) were significantly greater than in a group of nonathletes (37.78 mL/kg/min), and, in particular, aerobic athletes (like runners, cyclists, and skiers, excluding sprinters) showed higher values than anaerobic athletes (like sprinters and heavy weightlifters) (58.88 mL/kg/min vs. 52.04 mL/kg/min).

Increasing the VO_2max_, lowering the fat content, and increasing the ventilation are evidences of good adaptation to the effort and an increase in the cardiovascular efficiency [15]. Minute ventilation and lung diffusion capacity are factors related to the functioning of the respiratory system affecting the value of VO_2max_. In people with good physical fitness it is about 110–130 L/min, in sportsmen 150–160 L/min, and in some cases up to 200–210 L/min [16]. Its increase, as in these studies, indicates an improvement in exercise capacity and better oxygenation of the body during maximum effort.

Higher maximum values have been recorded [17]. The winners of Marcialonga, Vasaloppet, and Birkebeinerrennet (which are the three most prestigious long-distance ski races) also presented higher values [18]. Of course, it is not surprising, if we take into consideration the generally higher sports level of subjects and professional training. Elite skiers train mostly around 20–25 h per week during the preparation period from May to October [3]. In this study amateur skiers trained a mean of 41.7 h per month, which is 10.4 h per week. 

As a result, amateurs show lower maximum values for minute ventilation. Therefore, the tendency and increase in response to training should be evaluated. It also indicates a positive adaptation of the cardiovascular system to effort. 

Statistically significant differences were also shown in cases of the percentage and kilogram reductions of body fat. Studies have shown that this factor significantly affects VO_2max_ [19]. In other studies it was noted that skiers should aim to achieve a body composition with a high percentage of lean mass and a low percentage of fat mass. A focus on trunk mass through increased muscle mass appears to be important, especially for amateur and long-distance skiing, where double-poling is the most common technique [20,21]. Another research suggested that large amounts of lean body mass, especially in the arms, seem to be of great importance for cross-country skiing performance [22]. Elite male cross-country skiers have approximately 10.5% body fat [23]. In this research the level was higher: 15.3 ± 2.6 in the first test, and 14.6 ± 3.0 in the second. The level was similar to young non-athletes [24] but BMI was similar to athletes. Interestingly, the parameters were much better than those obtained in [8], where body fat mass in % was 23.4 ± 7.5 in the first test (before the training program) and 23.6 ± 7.0 in the second, after a six-month triathlon training program. BMI was 25.0 ± 2.7 and 24.7 ± 2.4, respectively.

In this study, a statistically significant decrease in body fat mass was recorded. Similar trends were also seen in pilot studies [25]. It was proven that a decrease in body fat mass can improve exercise capacity and results [26]. Optimal body fat for men in endurance disciplines is around 8–10% (during the competitive period) [27]. In [28] it was recommended that cross-country skiers should have 7% to 12% fat mass in the body. A reduction of body fat below 4%, however, can affect the body’s regenerative capacity and adversely affect the immune system. A value of >10% of body fat translates into poorer sports results because of, among other factors, higher than optimal body weight. By lowering body weight accordingly, by reducing body fat, we increase the level of oxygen intake per kilogram of body weight. Higher oxygen availability translates into better exercise options [27].

In addition, researchers highlighted the link between high body fat content and mortality. Reducing body fat instead of total weight seems to benefit the cardiovascular system more than a decrease in body mass index [29]. The results of these studies indicated the health impact of physical activity on the health of amateurs. A further decline may positively affect the effort and health of these amateurs but should not fall below 8%. Researchers suggested that a low lean mass index can be a strong indicator of mortality in men [30,31]. Reducing body fat and engaging in physical activity are important factors in improving the cardiovascular risk profile in middle-aged men. Achieving the correct body fat content can bring many health benefits for male amateur cross-country skiers.

The mean race intensity in the Vassaloppet race was 82% of maximal heart rate (HR) and did not differ between performance groups, even though elite skiers skied ∼15% faster than amateurs. The research showed that the amateur group contributed a longer effort in zones two and three, in comparison with elite cross-country skiers [32]. This emphasizes the role of aerobic and mixed possibilities in amateur efforts.

An increase in heart rate on lactate threshold (HR AT) was recorded in these studies. This indicates a positive adaptation to effort in response to a four-month workout. Delaying the transition from exercise in an aerobic to an anaerobic zone has a positive effect on exercise capacity. Shifting the lactate curve to the right results in a greater use of fat stores than of muscle glycogen, of which reserves in the body are limited.

During endurance training, the exercise load at the anaerobic threshold level is considered to be the most effective in relatively long exercises. Subsequent crossing of the lactate threshold during increasing work load allows you to extend your effort. When lactate increases, hydrogen ions are also released, which is the main cause of fatigue. A decrease in lactate concentration at the anaerobic threshold and in the maximum concentration will indicate correct adaptation to exercise. The optimal training of a long-distance skier should mainly approach the maximum oxygen consumption–VO_2max_ with the least accumulation of lactic acid in the blood [33]. Lowering the concentration of lactic acid and minimizing the effects of its secretion at higher speeds are important matters of training for long-distance runners. It is also worth noting that lactate shows a high correlation with other indicators, e.g., VO_2max_ [34].

The correct response to exercise was recorded in these studies. This was indicated by a decrease in lactate concentration at the threshold and in maximum values. However, it should be emphasized that a decrease in lactate concentration may also be an indicator of fatigue. A low-carbohydrate diet may be another factor affecting its lower values. Both of these factors will reduce the body’s exercise capacity.

## 5. Conclusions

The study indicates that a four-month comprehensive training for amateur long-distance skiers with the use of roller skis has a positive effect on the cardiovascular system. In addition, reducing body fat can be an important factor in protecting against heart disease in middle-aged men. A statistically significant increase was noted in maximum oxygen uptake and maximum minute ventilation, and a decrease in body fat content, maximum lactate concentration and lactate threshold, and heart rate on AT. Research indicated that in the amateur group increases similar to those in top competitors were achieved in the parameters tested, but the initial level was often significantly lower. Future research may focus on the analysis of a larger research group, include control groups, and focus especially on upper body training.

## Figures and Tables

**Table 1:** Comparison of the results of the first and second tests.

Parameter Mean (Standard Deviation)	Result 1	Result 2	*p*–Value
Height (cm)	182.2 (6.0)	182.2 (5.8)	1.000
Body weight (kg)	78.8 (6.3)	78.4 (6.0)	0.333
BMI (kg/height^2^)	23.7 (1.3)	23.6 (1.2)	0.191
% body fat mass	15.3 (2.6)	14.6 (3.0)	0.038
Body fat mass (kg)	12.1 (2.6)	11.4 (2.7)	0.023
Test duration (s)	1566.7 (130.4)	1592.7 (133.5)	0.267
Running speed (maximum km/h)	14.0 (0.7)	14.2 (0.8)	0.181
VO_2max_ (relative) (mL/kg/min)	49.0 (4.5)	51.1 (4.6)	0.008
VO_2max_ (absolute) (L/min)	3.9 (0.4)	4.0 (0.5)	0.054
Maximum heart rate (heartbeats per minute)	183.9 (9.8)	182.3 (10.1)	0.195
Maximum lactate concentration (mmol/L)	12.5 (2.8)	10.8 (2.5)	0.013
Maximum ventilation (L/min)	147.3 (20.5)	151.2 (22.2)	0.038
The frequency of maximum breathing (number of breaths/min)	59.7 (12.4)	60.4 (10.6)	0.410
Running speed anaerobic threshold (AT) (km/h)	11.1 (0.7)	11.2 (0.7)	0.575
Oxygen uptake–VO_2_ (relative) on AT (mL/kg/min)	44.8 (3.8)	44.9 (3.6)	0.934
Oxygen uptake–VO_2_ (absolute) on AT (L/min)	3.6 (0.4)	3.5 (0.4)	0.609
Heart rate on AT (number of breaths per min)	170.4 (7.7)	168.1 (10.4)	0.073
Lactate concentration (on the threshold) AT (mmol/L)	4.9 (1.3)	4.0 (0.9)	0.017
AT ventilation (L/min)	108.9 (17.9)	112.2 (18.6)	0.229
Heart rate on AT (bpm)	40.09 (6.2)	43.4 (7.1)	0.024
